# Automatic lumen detection and magnetic alignment control for magnetic-assisted capsule colonoscope system optimization

**DOI:** 10.1038/s41598-021-86101-9

**Published:** 2021-03-19

**Authors:** Sheng-Yang Yen, Hao-En Huang, Gi-Shih Lien, Chih-Wen Liu, Chia-Feng Chu, Wei-Ming Huang, Fat-Moon Suk

**Affiliations:** 1 Department of Electrical Engineering, National Taiwan University, Taipei, Taiwan; 2 Division of Gastroenterology, Department of Internal Medicine, Taipei Municipal Wan Fang Hospital, Taipei Medical University, No. 111, Section 3, Xing Long Road, Taipei, 116 Taiwan; 3 Department of Internal Medicine, School of Medicine, College of Medicine, Taipei Medical University, Taipei, Taiwan

**Keywords:** Biotechnology, Gastroenterology, Engineering

## Abstract

We developed a magnetic-assisted capsule colonoscope system with integration of computer vision-based object detection and an alignment control scheme. Two convolutional neural network models A and B for lumen identification were trained on an endoscopic dataset of 9080 images. In the lumen alignment experiment, models C and D used a simulated dataset of 8414 images. The models were evaluated using validation indexes for recall (R), precision (P), mean average precision (mAP), and F1 score. Predictive performance was evaluated with the area under the P-R curve. Adjustments of pitch and yaw angles and alignment control time were analyzed in the alignment experiment. Model D had the best predictive performance. Its R, P, mAP, and F1 score were 0.964, 0.961, 0.961, and 0.963, respectively, when the area of overlap/area of union was at 0.3. In the lumen alignment experiment, the mean degrees of adjustment for yaw and pitch in 160 trials were 21.70° and 13.78°, respectively. Mean alignment control time was 0.902 s. Finally, we compared the cecal intubation time between semi-automated and manual navigation in 20 trials. The average cecal intubation time of manual navigation and semi-automated navigation were 9 min 28.41 s and 7 min 23.61 s, respectively. The automatic lumen detection model, which was trained using a deep learning algorithm, demonstrated high performance in each validation index.

## Introduction

Colonoscopy is considered the gold standard for the detection of colorectal cancer. Screening colonoscopy significantly reduces colorectal cancer incidence and cancer-related mortality among individuals who undergo screening colonoscopy^1–4^. However, colonoscopy is an invasive examination; 16.7% of patients report moderate or severe abdominal pain after colonoscopy^5^, seriously hampering the successful completion of colon examinations.

Capsule colonoscopy was introduced in 2006 as a minimally invasive technique for examining the colon^6^. However, the movement of the capsule is passive, proceeding with the help of gastrointestinal tract peristalsis and gravity forces, which creates a large number of images that colonoscopists could spend a tremendous amount of time after the examination^7,8^. External controllability of a capsule colonoscope by means of an applied magnetic field is a possible solution to the maneuverability problem^9,10^. We have reported the feasibility and safety of a novel magnetic-assisted capsule endoscope system for the examination of the upper gastrointestinal tract^11,12^.

We further developed a magnetic capsule colonoscope (MCC) and magnetic-assisted capsule colonoscope (MACC) system based on a magnetic-navigated endoscope system. Compared with traditional colonoscopy, this MACC system is able to control the movement and orientation of the MCC by using a magnetic field navigator (MFN). Furthermore, the magnetic-assisted system is a promising locomotion methodology that has advantages in effectively navigating and posing during the diagnostic task^13,14^. Nevertheless, unknown front viewing angle, unpredictable rotation of capsule endoscope and unintuitive operation may cause confusion and inefficiency during operation^15,16^.

Several studies have used computer-assisted diagnosis (CAD), an artificial intelligence (AI) auxiliary system^17^, to assist gastroenterologists in performing colonoscopy. Object detection^17–21^ using AI and deep learning is a key computer vision division within the CAD system. With its speed and accuracy, the proposed lumen detection method can provide clues that can not only be used to reorient the MCC but also align it with the gastrointestinal tract in real time. Hence, we integrated a computer vision-based object detection and alignment control scheme into the MACC system.

In this study, we developed a lumen detection and alignment algorithm that enhances the efficiency of lumen identification and navigation of the capsule.

## Results

### Lumen detection: inferencing with the endoscopic dataset

**Figure 1:**
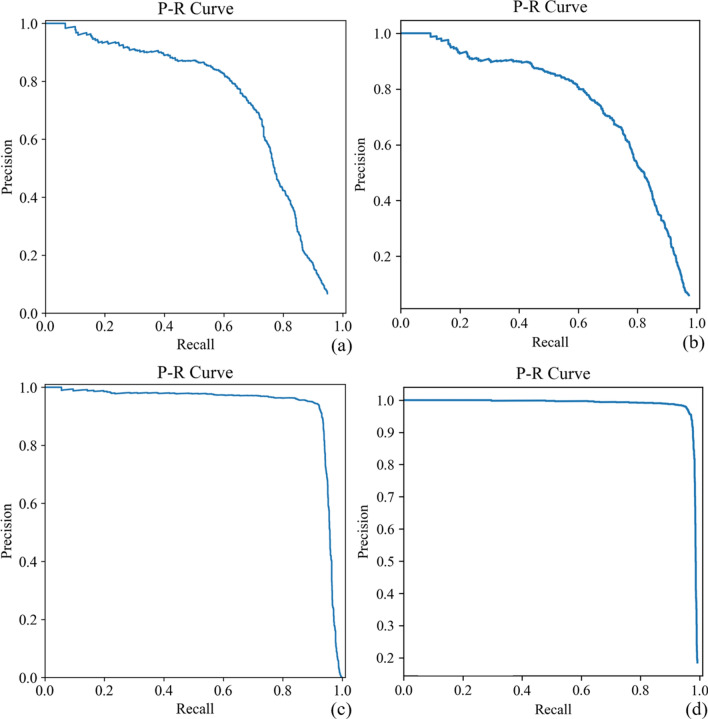
P-R curves for different models; **(a)** model A, **(b)** model B, **(c)** model C, and **(d)** model D. The AUC for models A, B, C and D were 0.718, 0.744, 0.935, and 0.982, respectively.

The purpose of this experiment was to inference lumen detection with endoscopic images. Two models, A and B, were used in this part. Table 1 presents the results of the IoU comparison. Model B had better testing results than model A. In inferencing by model B, when IoU was at 0.3, R was 0.678, P was 0.757, mAP was 0.614, and the F1 score was 0.715. The P-R curves for the experiments are depicted in Fig. 1. The areas under the P-R curves (i.e., Area under curve (AUC)) were 0.718 and 0.744 for models A and B, respectively. Model B was 3.62% better than model A in predictive performance.

**Table 1:** Results of the recall, precision, mAP, and F1 score in the lumen detection experiments for 4 models with images from endoscopic and simulated datasets.

Model	Dataset	Training images	Testing images	Validation index	IoU@0.3	IoU@0.5	IoU@0.7
A	Endoscopic dataset	7264	1816	P	0.756	0.750	0.683
				R	0.673	0.676	0.676
				mAP	0.611	0.611	0.604
				F1	0.712	0.711	0.680
B	Endoscopic dataset (without negative sample)	3149	787	P	0.757	0.582	0.257
				R	0.678	0.521	0.230
				mAP	0.614	0.421	0.113
				F1	0.715	0.550	0.242
C	Simulated dataset	7147	1788	P	0.904	0.878	0.864
				R	0.865	0.884	0.869
				mAP	0.838	0.895	0.835
				F1	0.884	0.881	0.866
D	Simulated dataset (without negative sample)	6731	1683	P	0.961	0.925	0.762
				R	0.964	0.928	0.764
				mAP	0.961	0.908	0.672
				F1	0.963	0.926	0.763

### Lumen detection: inferencing with the simulated dataset

We implemented the testing on models C and D with and without negative samples. The purpose of these models was for lumen alignment experiments in the MACC system. Model D outperformed model C by approximately 5% in every validation index (Table 1). R tested by model D was 0.964, P was 0.961, mAP was 0.961, and the F1 score was 0.963 when IoU was at 0.3. The P-R curves for the experiments for models C and D are shown in Fig. 1c,d. The AUCs of the P-R curves for models C and D were 0.935 and 0.982, respectively. Model D outperformed models A, B, and C by 36.77%, 31.99%, and 5.03%, respectively.

### MCE alignment control using the simulated dataset

**Figure 2:**
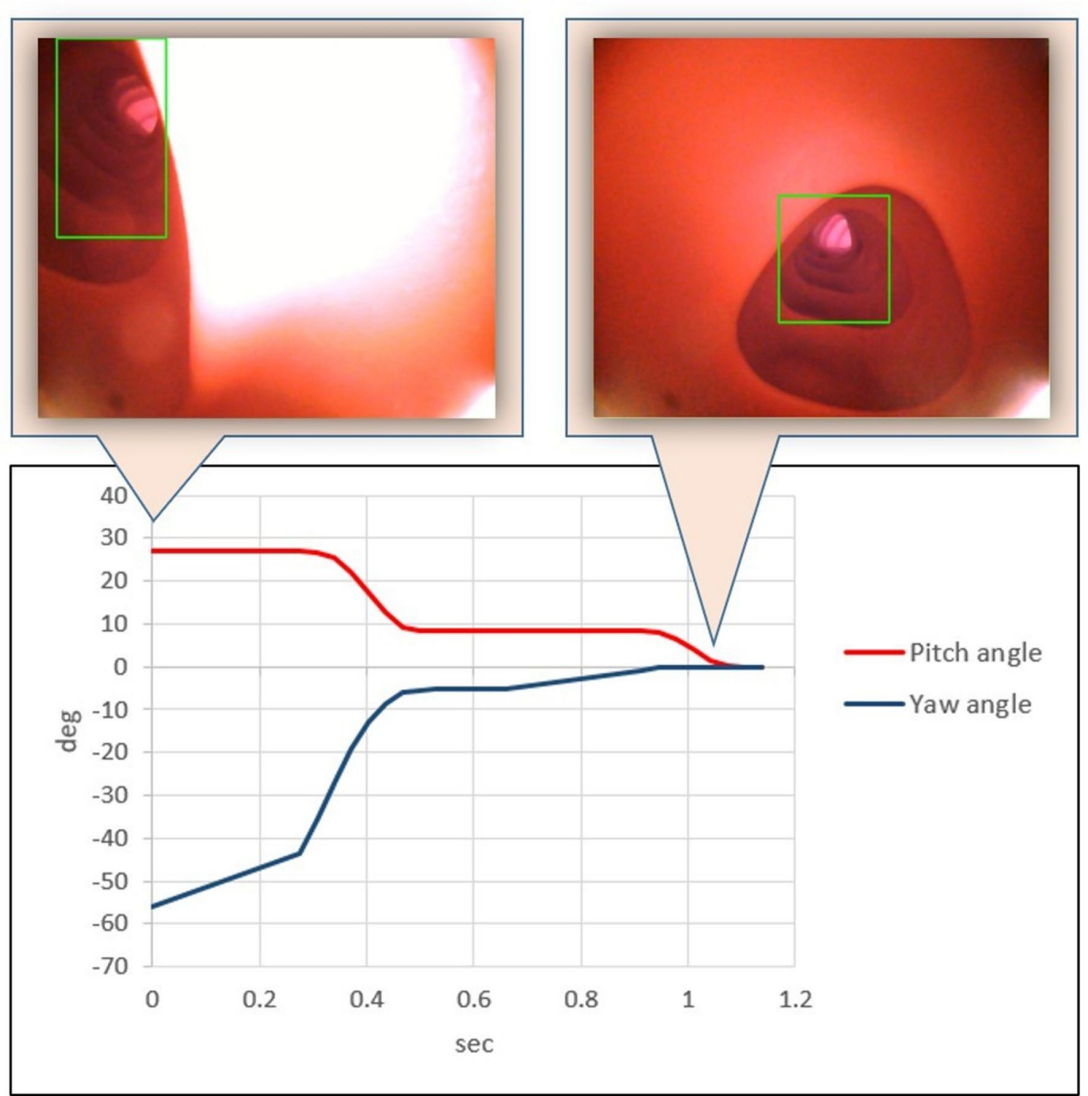
Record of the MCC rotated by angles of yaw and pitch. The position of the lumen was initially located in the northwest area of the image. The MFN completed the alignment in 1.14 s with the PI control. deg, adjustment of yaw and pitch in degrees; sec, time required to adjust the angle in seconds.

Model D demonstrated the best R, P, mAP, and F1 score. Therefore, we applied model D to the MFN for the lumen alignment experiment. Figure 2 depicts a single alignment test. The MFN rotated the pitch and yaw angles of the MCC by 26.89° and 56.16°, respectively. The procedure required 1.14 s. Then, experiments were conducted in 8 directions, and each direction was performed 20 times for a total of 160 trials. Detailed results are listed in Table 2. The mean degrees of adjustment for yaw and pitch of the MCC were 21.70° and 13.78°, respectively. The mean alignment control time of the trials was 0.902 s.

**Table 2:** Results of yaw and pitch adjustments in lumen alignment experiments with 8 directions using model D.

Orientation^a^	Yaw adjustment^b^	Pitch adjustment^b^	Time consume^c^
A	27.15 ± 4.97	19.95 ± 4.72	0.978
B	0.10 ± 0.30	23.15 ± 3.07	0.750
C	33.70 ± 8.13	4.35 ± 5.67	1.140
D	35.50 ± 2.85	0.90 ± 1.18	0.756
E	27.65 ± 1.85	13.85 ± 1.93	0.836
F	29.80 ± 4.23	9.95 ± 4.88	1.018
G	0.75 ± 0.77	17.75 ± 1.55	0.742
H	18.95 ± 7.63	20.30 ± 6.36	0.999
160 trials	21.70 ± 13.95	13.78 ± 8.58	0.902

^a^Eight starting positions were designed and 20 trials were performed for each position for a total of 160 trails; A. northwest B. north C. northeast D. east E. southeast F. south G. southwest H. west.

^b^Degree of adjustment of yaw angle and pitch angle expressed as mean ± standard deviation.

^c^Time spent adjusting yaw and pitch angles for each position, expressed as mean time in seconds.

### Intubation time of manual navigation and semi-automated navigation

**Figure 3:**
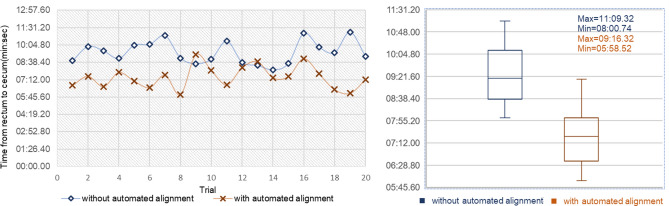
Intubation time from the rectum to cecum. Manual navigation and semi-automated navigation were represented by blue and brown lines, respectively. The average cecal intubation time of manual navigation and semi-automated navigation were 9 min 28.41 s and 7 min 23.61 s, respectively.

To confirm the performance of the MACC system, we performed 20 trials of manual navigation and semi-automated navigation in the same colonoscopy training stimulator. The alignment rates of the automated alignment system at the rectum, sigmoid, descending, transverse and ascending colon were 10.61%, 95.45%, 76.47%, 84.31% and 86.11%, respectively. The average cecal intubation time of manual navigation was 9 min 28.41 s. In semi-automated navigation, the average cecal intubation time was 7 min 23.61 s. The cecal intubation time of semi-automated alignment was 21.96% lesser than that of manual navigation**.** Detailed results are shown in Fig. 3.

## Discussion

Common applications of AI to endoscopy are for the detection and analysis of inflammatory lesions, polyps, and cancer. Detection of gastrointestinal bleeding is the most common application in capsule endoscopy^17^. The concept for lumen detection with AI has been proven in several studies^18,22,23^. In this study, we used 2 image datasets to train 4 deep learning-based lumen detection models. We presented the performance of models A and B trained by an endoscopic dataset and models C and D trained by a simulated dataset. Our approach consisted of 2 steps. First, we developed 4 CNN models for locating the lumen. Second, we took the center of the predicted bounding box as a reference position during the testing phase in the lumen alignment experiment. Once the reference position was acquired, the MACC system aligned it to the center of the screen (Supplementary video). In a comparison of the AUC of P-R curves, the predictive performance of model D was better than that of models A, B, and C by 36.77%, 31.99%, and 5.03%, respectively. We then applied model D to an alignment experiment with the MACC system. MCC alignment was controlled in 8 directions by using a strong radial magnetized permanent magnet on the MFN.

Overfitting is a common concern in deep learning and statistics. It occurs when a constructed model excessively matches a training dataset but goes wrong with other external testing datasets^24,25^. To prevent overfitting, we implemented several techniques when training the models, such as data augmentation, BN, and weight decay. The application of BN has several benefits, such as removing dropout and accelerating learning rate decay, that make networks train faster^26,27^. Previous studies^28,29^ have determined that weight decay is a regularizer that avoids overfitting; it also reduces the square error during training. Restated, penalizing the neural network during training according to the weights of the network minimizes overfitting.

Regarding the object detector, two-stage detectors, such as R-CNN^30^, Fast R-CNN^31^, Faster R-CNN^32^, and Mask R-CNN^33^, use a region proposal network to produce regions of interests in the first stage. In the second, the region proposals for object classification and bounding box regression are sent. By contrast, one-stage detectors, such as YOLO v1-v3^34–36^ and the singe shot multiBox detector^37^, treat object detection as a regression problem and skip the region proposal stage to detect directly. Because of its one-stage design, the one-stage detector is generally superior to the two-stage detector in inference speed but suffers in detection accuracy. However, YOLO v3 not only outperforms other conventional one-stage object detectors in speed but also compares with two-stage object detectors in accuracy. In addition, the model architecture of YOLO v3 uses Darknet-53 instead of Darknet-19 as the feature extractor. Darknet-53 requires fewer floating-point computations, making calculation more efficient and prediction faster. Furthermore, YOLO v3 uses multi-label classification and independent logistic classifiers for better performance than softmax. It uses binary cross-entropy loss to give normalized probabilities to predict class during the training process. In our experiment, however, we labeled only the region of the lumen for single class detection. We evaluated the model by P-R curve because no negative label was present in any image in our training set.

In a previous study, Zabulis et al. detected the lumen by the mean shift algorithm^18^. The algorithm runs several times with various data points. For each point, the mean shift defines a region around it and computes the mean of data points. Then, it shifts the center of the region to the mean and repeats this process until it converges. In the end, the region shifts to a denser place of the dataset. In their experiments, the frame rate was 0.33 fps, which is not fast enough to apply to a video during colonoscopy. However, our proposed method inferenced on the MCC with an average rate of 30 fps. Gallo et al. proposed a boosting classification-based method for lumen detection^38^. Their best classification result for R and P were approximately 0.9 and 0.7, respectively. Wang et al. utilized Bayer-format downsample, adaptive threshold segmentation, and radial texture detection techniques to identify the intestinal lumen. The precision and sensitivity of lumen detection were reported as 95.5% and 98.1%, respectively^23^. The speed was 0.02 s per frame, but they used low resolution images (64 × 64) to reduce computation complexity. In our results, precision and sensitivity were 96.1% and 96.4%, respectively, even at a high resolution (1920 × 1080). With YOLO v3, inference speed was 0.033 s per frame.

Two navigation scenarios have been designed to prove the effectiveness and feasibility of the MACC system. In semi-automated navigation, the MACC system manipulated the MFN based on the integration of automated alignment system. Although this system can perform automatically align, there were two situations that might need the operators to intervene during intubation process. The first situation was the lumen image with unclear contour because of the lubricant sticking on the camera. In the second situation, the alignment rate was poor when capsules passed through a sharp angle, which most frequently occurred at the rectosigmoid junction.

This study has several limitations. First, the models trained on the endoscopic dataset were less precise than those trained on the simulated dataset. The reason for this may be that the endoscopic dataset contained many lumen images with unclear contours or even with contours covered by stool, mucus, or bubbles, making it more difficult for the neural network to extract the features it was supposed to learn. Second, the variation of endoscopic images was higher than we expected. A possible solution for this may be additional data cleaning, scrubbing, and augmentation. Removing similar images from the endoscopic dataset and relabeling the lumen as the proper region could solve the problem. Finally, a clinical trial is required to prove that this MACC system with automatic lumen alignment shortens cecal intubation time.

## Conclusion

This study used a deep learning algorithm and automatic lumen detection model that demonstrated high precision and recall with endoscopic and simulated datasets. Coordinating the lumen detection model with alignment control, this integrated method may increase the performance and efficiency of capsule colonoscopy. The MACC system has promise for increasing the navigation efficacy of capsule colonoscopy.

## Methods

### MACC system

**Figure 4:**
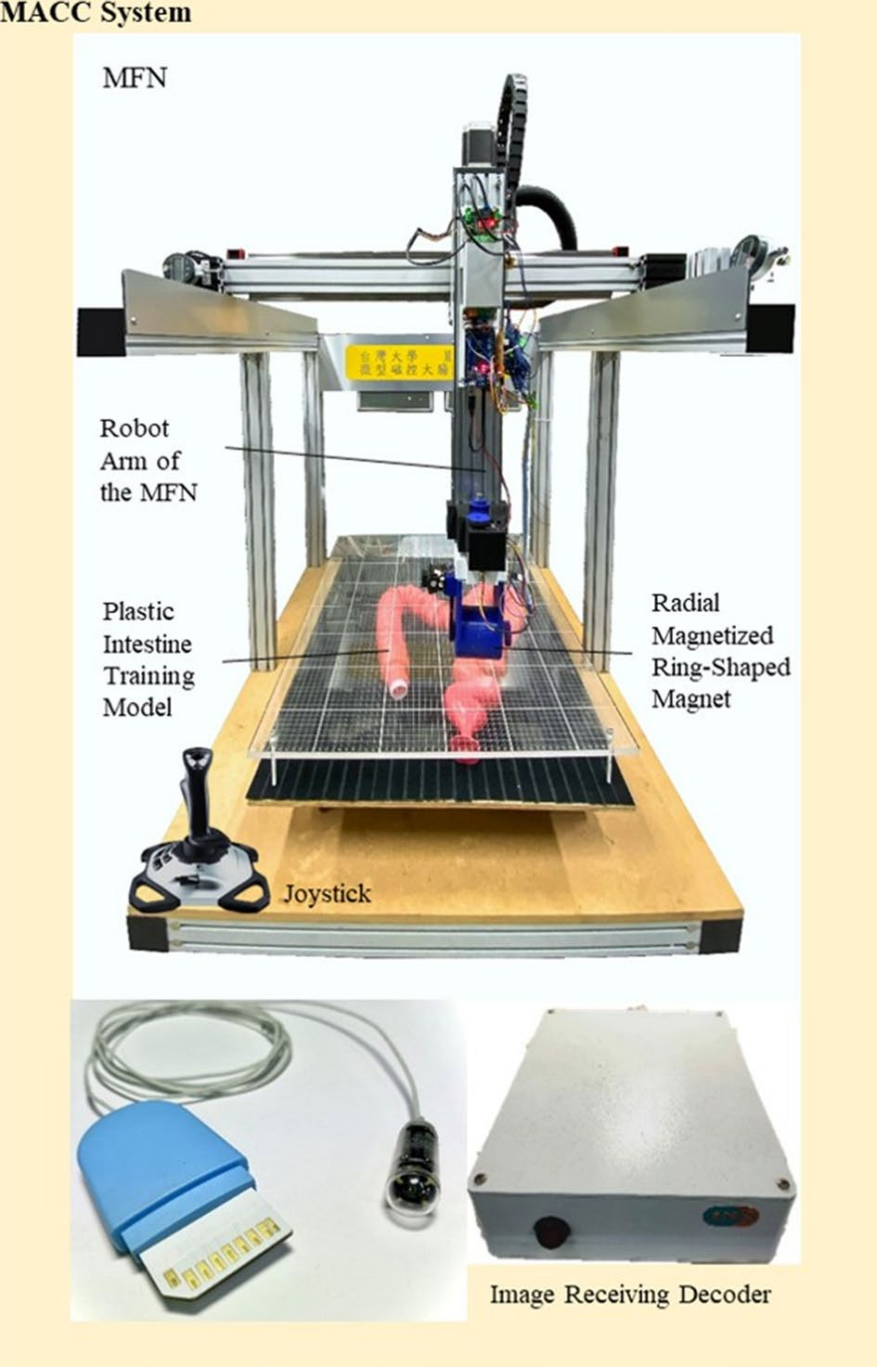
Apparatus of the MACC system, consisting of the MFN, MCC, image receiving decoder, and joystick.

The proposed MACC system consists of an MFN, MCC, image receiving decoder, and joystick (Fig. 4). The MFN is capable of 5 degrees of freedom operation with a working space of 650 × 650 × 410 ${\mathrm{mm}}^{3}$. The MFN has a radial magnetized ring-shaped magnet (NdFeB alloy) driven by a servo motor through the belt to locomote the MCC inside the colon lumen. The MCC measures 25.5 mm × 9.9 mm and weighs 4.64 g. Its components were described elsewhere^11^. Briefly, it has an internal permanent magnet, 4 white light-emitting diodes, optical modules, including a lens and complementary metal-oxide semiconductor (CMOS) sensor, and a thin cable. Images are transmitted at 30 frames per second (fps) from the CMOS sensor, and the image resolution is 640 × 480 pixels. An Extreme 3D Pro Joystick (Logitech International S.A., Lausanne, Switzerland) is utilized to control the movement and direction of the MFN.

### Colonoscopy image dataset

This study was approved by the Joint Institutional Review Board of Taipei Medical University. Owing to the retrospective review of colonoscopic images in this study, the Taipei Medical University ethics committee waived the need for patient informed consent. All methods were carried out in accordance with relevant guidelines and regulations. Two datasets were used in this study. The endoscopic dataset without any information of patients and the simulated dataset from a colonoscopy training model simulator (Kyoto Kagaku Co., Ltd., Japan), a previously validated physical model simulator^39^. The endoscopic dataset contained a total of 9080 colonoscopic images. In all, 3934 images contained sight of lumen. The remaining 5146 were either unrecognizable or concealed as negative samples for lumen detection. We split the 9080 images into the training and testing sets randomly with a ratio of 4 to 1. The simulated dataset comprised 8414 images in total. We randomly chose 6731 images for training and 1683 images for testing for a ratio of 4 to 1.

Models A and B were trained by the endoscopic dataset. The purpose of these models was to prove that our lumen detection method can actually work well with real endoscopic images. Models C and D used the simulated dataset taken from the simulator. We chose the best model to inference as the input for the lumen alignment experiment in the MACC system. We trained and tested models A and C with images that included the negative samples, which means there could be no instance of the lumen in partial images. By contrast, models B and D were trained and tested with images without negative samples, which means, in every image, at least one instance of the lumen existed. All 4 models used the same parameter settings and personal computer to train. No training images would be used as inference sources.

We implemented several techniques while training the model, including data augmentation, batch normalization (BN), and weight decay, to prevent overfitting. BN is a method we can use to normalize the inputs of each layer. A BN layer has a regularizing effect similar to that of a dropout layer but makes networks train faster. Weight decay is another technique used to avoid overfitting by limiting the size of neuron weights. For data augmentation, images were flipped vertically and horizontally, and each image was randomly variated with salt-and-pepper noise and Gaussian noise to enhance the robustness of models.

### Training and testing of the convolutional neural network model

All experiments were operated with Python 3.6.8 and Pytorch 1.3.1. Python is an interpreted, object-oriented, high-level dynamic programming language. Pytorch is a python package that provides tensor computation with graphics processing unit (GPU) acceleration. All models were trained and evaluated on a personal computer with an Nvidia GTX 1080Ti GPU (NVIDIA Corporation, Santa Clara, CA, USA) with 11 GB of total memory. The operating system was Windows 10. Using transfer learning, the models loaded a trained weight file that was pretrained on ImageNet to initialize them. ImageNet is a large visual database intended for research on visual object recognition. The 4 models were developed based on the You Only Look Once version 3 (YOLO v3), an end-to-end convolutional neural network (CNN) able to make inferences of multiple rectangle box locations and classes. Restated, YOLO v3 allows one-stage, simultaneous object detection and localization. With regard to bounding box prediction, YOLO v3 uses dimension clusters as an anchor box to predict 4 coordinates for each bounding box border. The labeling work was performed by 4 investigators (Chu, HE Huang, WM Huang, and Yen), and all annotations were confirmed by an experienced gastroenterologist (Suk).

**Figure 5:**
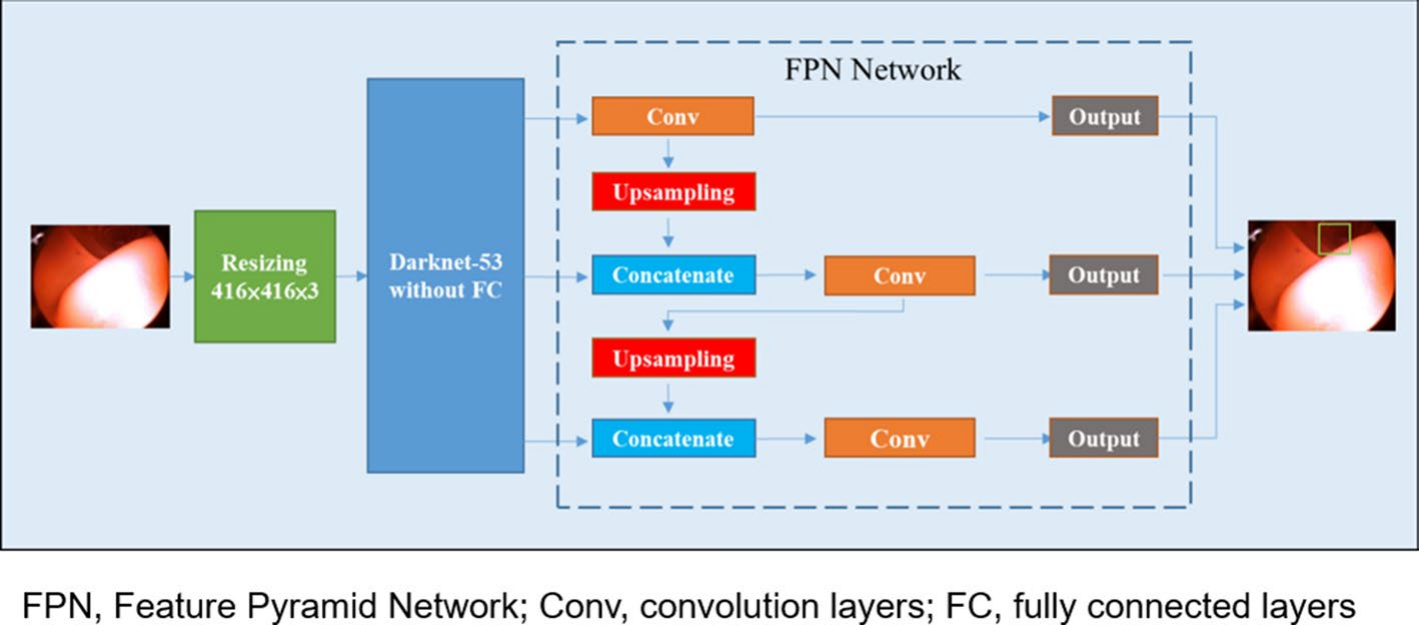
Workflow and model architecture of lumen detection. The input was the endoscopic image. The neural network inferenced the presence and position of the lumen. The output was the image with the bounding box as the detection of the lumen. *FPN* Feature Pyramid Network, *Conv* convolution layers, *FC* fully connected layers.

In preprocessing, we saved the bounding box of the lumen as ground truth in text files. The text files were read during the training process. Next, the model parameters were updated by a stochastic gradient decent (SGD) optimizer according to training loss. The binary results generated from the SGD optimizer were classified as the presence of the lumen if any value was > 0.5. The network learning rate was 0.001 in our experiments and was tuned using exponentially decaying weights (0.0005). This method effectively suppressed model overfitting. The batch size was 4, and training stopped when the epoch was greater than 150. We used the nonmaximum suppression (NMS)^40^ method with predicted results to remove redundant bounding boxes to find the best location for object prediction. Finally, the best lumen detection bounding box on the original image was added. The developed model architecture for lumen detection is presented in Fig. 5.

### Magnetic lumen alignment control

**Figure 6:**
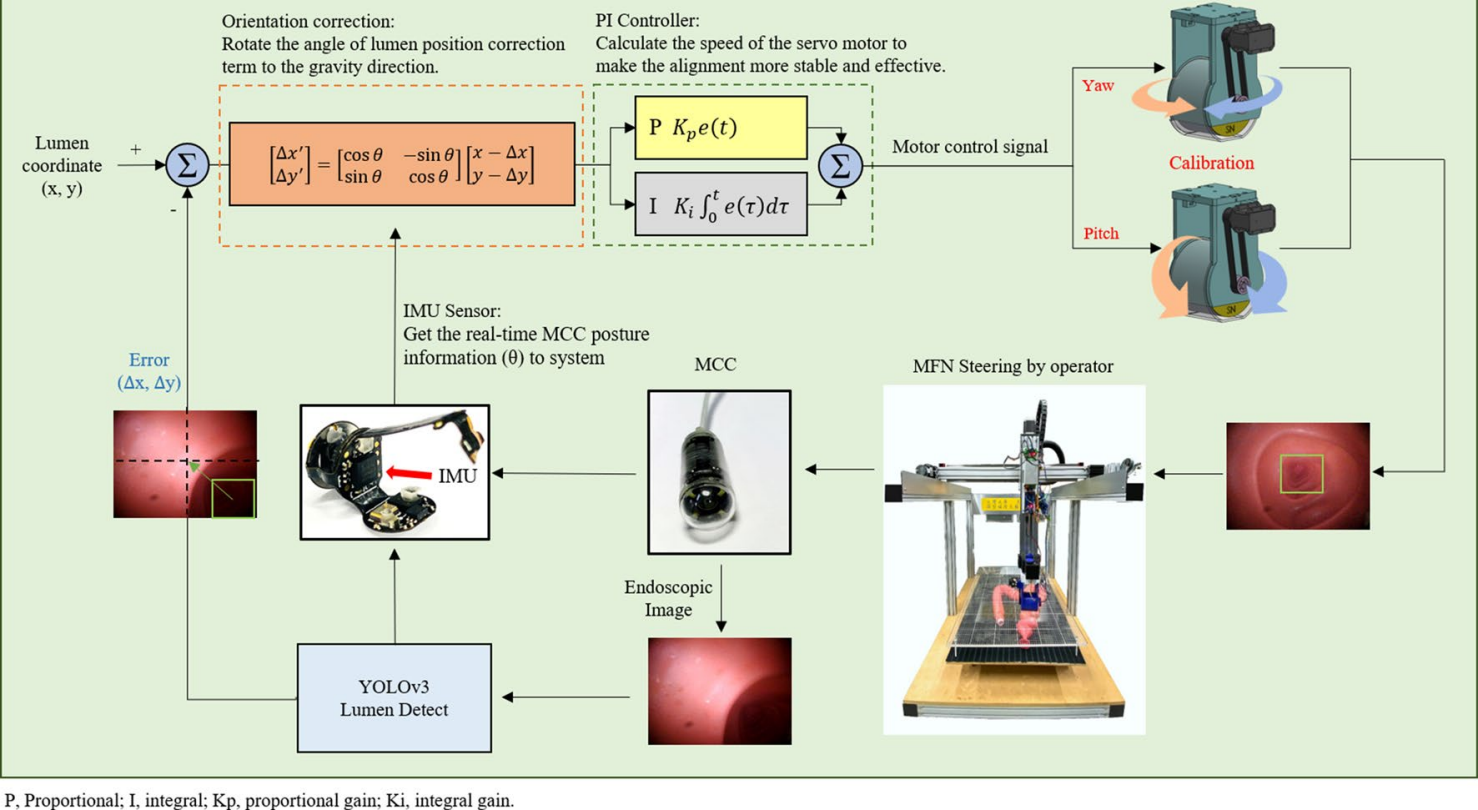
Control block diagram for alignment experiment with the MACC system. Using the calibration error between the lumen position and the image center, real-time posture information of the MCC was sent to the orientation correction and PI controller. The MFN calibrated by yaw angle and pitch angle according to instructions given by control signal from PI controller until the calibration error reached the predefined threshold. *P* proportional, *I* integral, *Kp* proportional gain, *Ki* integral gain.

An overview of the alignment control framework is depicted in Fig. 6. We read out the coordinate of the bounding box center and used it to calculate the calibration error with the center of the image. The calibration error was defined as the Euclidean distance between the bounding box center and image center. According to real-time posture angle values measured by an inertial measurement unit sensor (Freescale Semiconductor, Tempe, AZ, USA) constructed inside the MCC, we sent the posture angle to rotating matrix to maintain absolute horizon in camera for the sake of surgeon’s performance. Then, the results were sent to the proportional integral (PI) controller to manipulate the servo motor on the MFN. Use of PI control was for the purpose of quick minimization of alignment bias (steady-state error). Then, the MFN calibrated to adjust the yaw and pitch angles on the basis of instructions given by the PI controller. The alignment stopped when calibration error was smaller than 50 pixels (1 pixel = 0.265 mm), and our image resolution was 640 × 480 pixels. We implemented the alignment control for 160 trials in 8 orientations (north, northeast, east, southeast, south, southwest, west and northwest) and recorded the calibration time for each trial.

### Semi-automated navigation and manual navigation

In semi-automated navigation, lumen alignment was controlled by the automated alignment system during the navigation from the rectum to cecum. During the navigation, automated alignment system took fully control to reorient the MCC to the right direction instead of manually operation. If the capsule was struck in the lumen, operators might need to interfere optionally. To compare with semi-automated navigation, we performed manual navigation in the same colonoscopy training simulator. In this system, we used the joystick to control the MFN to navigate the MCC from rectum to cecum.

### Statistical analysis

The prediction was considered true positive (TP) if the area of overlap/area of union (IoU) was greater than the predefined IoU threshold. Otherwise, the prediction was a false positive (FP). That is, the model was confident of adding the bounding box at the correct place and moment on the image in the model testing phase, and the lumen was actually occurring in the image. The prediction was a false negative (FN) if the model failed to confirm the presence of a lumen when it actually occurred in the image.

The models were evaluated using several validation indexes^41^. Recall (R) was defined as the proportion of actual positives identified correctly (R = TP/[TP + FN]). Precision (P) was defined as the proportion of positive identifications actually correct (P = TP/[TP + FP]). Average precision (AP) was defined as the area under the P-R curve. The mean average precision (mAP) was the average of the AP for all classes; this was used to evaluate the precision of bounding box localization. The F1 score ([2 × R × P]/[R + P]) was a measure of a test’s accuracy. Its purpose was to balance P and R. The intersection over union (IoU = area of overlap/area of union) was defined as the overlap between the predicted bounding box and the ground truth bounding box. Therefore, IoU was a parameter used to test how accurately the boundary box was drawn in relation to ground truth. The P-R curve is a graph with y- and x-axis values for P and R. The curve indicates the trade-off between P and R for different thresholds and represents whether a data point was recognized in the positive class. AUC was used in the classification evaluation to determine which models best predicted the classes; a high AUC reflected high R and P. All statistical analyses were conducted using MATLAB 2019a numerical software.

### Ethical approval

This study was approved by the Joint Institutional Review Board of Taipei Medical University.

## Supplementary information

Supplementary video.

Supplementary video legend.

## Abbreviations

AI: Artificial intelligence
AP: Average precision
AUC: Area under curve
BN: Batch normalization
CAD: Computer-assisted diagnosis
CMOS: Complementary metal-oxide semiconductor
CNN: Convolutional neural network
FN: False negative
FP: False positive
fps: Frames per second
GPU: Graphics processing unit
IoU: Area of overlap/area of union
mAP: Mean average precision
MCC: Magnetic capsule colonoscope
MACC: Magnetic-assisted capsule colonoscope
NMS: Nonmaximum suppression
MFN: Magnetic field navigator
P: Precision
PI: Proportional integral
R: Recall
SGD: Stochastic gradient decent
TP: True positive
YOLO v3: You Only Look Once version 3
