# Supplementary material for: Automatic lumen detection and magnetic alignment control for magnetic-assisted capsule colonoscope system optimization
Source: Sci Rep. 2021 Mar 19;11:6460. doi: 10.1038/s41598-021-86101-9 (PMC7979719; doi:10.1038/s41598-021-86101-9)
Supplement: Supplementary file 2 — Supplementary video legend. [file 41598_2021_86101_MOESM2_ESM.docx]

**Video legend**

Video of alignment control recorded with the MACC system. The box at the upper-right corner is a synchronized alignment control of the MCC view. At the beginning, the lumen deviates from the center of the screen. The alignment control returned the view of the MCC to the center of the lumen
